# Does Laser Surgery Interfere with Optical Nerve Identification in Maxillofacial Hard and Soft Tissue?—An Experimental *Ex Vivo* Study

**DOI:** 10.3390/s151025416

**Published:** 2015-10-01

**Authors:** Bastian Bergauer, Christian Knipfer, Andreas Amann, Maximilian Rohde, Katja Tangermann-Gerk, Werner Adler, Michael Schmidt, Emeka Nkenke, Florian Stelzle

**Affiliations:** 1 Department of Oral and Maxillofacial Surgery, Friedrich-Alexander University Erlangen-Nuremberg (FAU), Erlangen 91054, Germany; E-Mails: christian.knipfer@fau.de (C.K.); andi.nbg@hotmail.de (A.A.); maximilian.rohde@fau.de (M.R.); florian.stelzle@fau.de (F.S.); 2 Bavarian Laser Center GmbH (blz), Erlangen 91054, Germany; E-Mails: k.tangermann@blz.org (K.T.-G.); michael.schmidt@lpt.uni-erlangen.de (M.S.); 3 SAOT—Graduate School in Advanced Optical Technologies, Friedrich-Alexander University Erlangen-Nuremberg (FAU), Erlangen 91054, Germany; 4 Department of Medical Informatics, Biometry and Epidemiology, Friedrich-Alexander University Erlangen-Nuremberg (FAU), Erlangen 91054, Germany; E-Mail: werner.adler@imbe.med.uni-erlangen.de; 5 Chair of Photonic Technologies, Friedrich-Alexander University Erlangen-Nuremberg (FAU), Erlangen 91054, Germany; 6 Department of Oral and Maxillofacial Surgery, Medical University of Vienna, Vienna 1090, Austria; E-Mail: emeka.nkenke@meduniwien.ac.at

**Keywords:** diffuse reflectance spectroscopy, laser ablation, laser surgery guidance, remote optical measurement, spectra analysis, remote surgical methods, optical nerve identification

## Abstract

The protection of sensitive structures (e.g., nerves) from iatrogenic damage is of major importance when performing laser surgical procedures. Especially in the head and neck area both function and esthetics can be affected to a great extent. Despite its many benefits, the surgical utilization of a laser is therefore still limited to superficial tissue ablation. A remote feedback system which guides the laser in a tissue-specific way would provide a remedy. In this context, it has been shown that nerval structures can be specifically recognized by their optical diffuse reflectance spectra both before and after laser ablation. However, for a translation of these findings to the actual laser ablation process, a nerve protection within the laser pulse is of utmost significance. Thus, it was the aim of the study to evaluate, if the process of Er:YAG laser surgery—which comes with spray water cooling, angulation of the probe (60°) and optical process emissions—interferes with optical tissue differentiation. For the first time, no stable conditions but the ongoing process of laser tissue ablation was examined. Therefore, six different tissue types (nerve, skin, muscle, fat, cortical and cancellous bone) were acquired from 15 pig heads. Measurements were performed during Er:YAG laser ablation. Diffuse reflectance spectra (4500, wavelength range: 350–650 nm) where acquired. Principal component analysis (PCA) and quadratic discriminant analysis (QDA) were calculated for classification purposes. The clinical highly relevant differentiation between nerve and bone was performed correctly with an AUC of 95.3% (cortial bone) respectively 92.4% (cancellous bone). The identification of nerve tissue against the biological very similar fat tissue yielded good results with an AUC value of 83.4% (sensitivity: 72.3%, specificity: of 82.3%). This clearly demonstrates that nerve identification by diffuse reflectance spectroscopy works reliably in the ongoing process of laser ablation in spite of the laser beam, spray water cooling and the tissue alterations entailed by tissue laser ablation. This is an essential step towards a clinical utilization.

## 1. Introduction

Laser surgery provides many advantages like marginal mechanical trauma and controllable coagulation [1,2,3,4,5,6]. Nevertheless, there exists a huge drawback in laser surgery. Other than using a scalpel or a rotary instrument, the surgeon gets no haptic information about the exact laser penetration depth or the currently ablated anatomical structure on the bottom of the crater. The ablation process has to be surveilled visually, which demands a high level of experience and skills. The use of lasers in surgery is therefore limited to the superficial ablation of tissues like the treatment of skin, superficial ablation of pathologies within the oral and glossopharyngeal tract, in ophthalmology (to create a corneal flap) or in gynecology [7,8,9,10,11]. If deeper tissue layers have to be ablated or in cases where the anatomy of the operation area is complex, the use of lasers always involves the risk of iatrogenic damage or destruction of critical anatomical structures that should be preserved (e.g., nerves or blood vessels). When focusing on the head and neck area, there are several nerves that are essential for both sensory or motor function and aesthetics. These structures are fundamental for the patients’ quality of life and have to be protected when performing laser surgery [12,13,14,15,16]. A regularly performed surgical procedure in the area of oral and maxillofacial surgery is the sagittal split osteotomy, which is used to correct the position of the mandible. A sagittal cut of the bone enables a repositioning of the lower jaw in dorso-ventral direction. Finally, the mandible can be fixed with plates or screws in the new position. Against the background of being a frequent surgical procedure in the area of head and neck, particularly the sagittal split osteotomy of the lower jaw often entails the risk of nerve damage. More than 70% of patients have some degree of neurosensory deficit one year post-surgery [17,18,19]. A trauma to the lower alveolar nerve causes a temporary or even permanent numbness of the lower lip which might impair the food intake. Furthermore, the removal of tumors of the salivary glands—which occur with a prevalence of 15.4 and an incidence of 2.51 per 100,000—inherits the risk of nerve damaging [20]. The majority of salivary gland tumors (65%) is located in the parotid gland within which the facial nerve branches out into five segments [21]. At deep localizations of malignant neoplasms an iatrogenic injury to this nerve was reported in up to 50% of the cases [15]. This can induce a loss of function of the ipsilateral mimic muscles. For a potential application of laser surgical methods in these widely performed surgical procedures, an automatic feedback mechanism for laser surgery that is able to identify the different biological tissue types on the one hand and that is able to stop the laser if the anatomical structure should be preserved on the other hand has been long demanded. For that purpose, the diagnostic process of the feedback loop must be fast and provide information in real-time as it has to be repeated with every laser pulse. For that purpose, remote optical tissue differentiation methods are particularly suitable. They are noninvasive, straightforward and cost-efficient. In particular, diffuse reflectance spectroscopy (DRS) presents a promising approach as spectra of different tissue types are already well-investigated and can be identified by certain spectral parameters within a short period of time and without the need of touching the sample (e.g., *ex vivo*: fatty tissue, bladder, brain, heart, kidney, liver, lung, muscle, skin, uterus and vessels; *in vivo*: brain, muscle and liver) [22,23,24]. The distinctive spectra occur due to the applied light which is absorbed or scattered, depending on the optical properties of each tissue type. In the visible range of the electromagnetic spectrum (approx. 390–700 nm) the main absorbers are melanin and hemoglobin. The main scatterers are cell organelles (such as mitochondria *etc.*) and the cells themselves [25]. It has yet been demonstrated that DRS is able to differentiate normal/benign tissue from pre-malignant and malignant tissue [26,27,28]. Similar results were found for the diagnosis of pathologically altered tissue respectively physiological cardiovascular tissue [29,30]. In this context our workgroup has establish an experimental measurement setup for the differentiation of various tissues of head and neck as a solid basis for a feedback loop for tissue specific laser surgery. In this part of the project, the general feasibility of optical tissue differentiation by diffuse reflectance spectroscopy (DRS) was demonstrated on five different tissue types in an *ex vivo* setting [31]. Additionally, it was shown that DRS is an adequate technique for nerve identification in the vicinity of bone and salivary gland [32,33]. In a second step, the viability of tissue identification was proven after the tissue was altered by laser ablation, focusing on nerve detection [33,34]. In a third step, the promising *ex vivo* results could be successfully transferred to an *in vivo* setup in rats [35]. Therefore, after investigating the stable conditions which prevail in pre and post laser exposure measurements (offline ablation) in previous publications of our working group [31,32,33,34,35], we addressed to the implementation of tissue differentiation during the dynamic process of laser ablation (online irradiation) as a final step of our *ex vivo* research. Despite the promising results of our workgroup in tissue differentiation pre as well as post laser ablation, the viability of DRS-based tissue differentiation in an online irradiation system for a closed laser surgery feedback mechanism still remained uncertain. Moreover, in order to ensure an adequate feedback loop of tissue identification even within the online laser surgical process, it is essential to investigate the proposed system for its ability for tissue identification despite the influence of the laser beam, the spray water cooling and the optical process emissions caused by the ongoing process of laser ablation in biological tissue.

To our knowledge, this research question has not been addressed in the context of online irradiation and DRS-based nerve identification in the head and neck area yet. For this reason, this work—as a base for further *in vivo* research—takes a crucial step for transferring the set-up into a clinical practicable remote feedback system for tissue-specific laser surgery that protects sensitive structures from iatrogenic damage.

## 2. Experimental Section

### 2.1. Tissue Samples

Diffuse reflectance spectra were measured on six different *ex vivo* tissue types obtained from 15 bisected domestic pig heads. To avoid any impact from pathological conditions only animals free from local or systemic diseases were considered in this study. Soft tissue was taken from the midfacial region. Nerve tissue from the infraorbital nerve, skin from the buccal region, muscle from the masseter muscle (without fascia) and fat from the subcutaneous fatty tissue of the buccal region were dissected carefully with a scalpel. The hard tissue (cortical and cancellous bone) was provided from the lower jaw. A raspatory and a micro jigsaw with high flow water cooling (100 mL/min, normal saline solution) were used to prepare the bone. All tissue samples were cut to a size of 5 × 5 cm and a thickness of 7–10 mm. Solely nerve tissue was—due to its anatomical structure—prepared with a length of 5 cm and an average diameter of 1 cm. To clean the tissue samples from all superficial contamination (clotted blood particles, *etc.*) they were carefully rinsed with sterile saline solution. The tissue was handled with care in order to avoid mechanical alterations of the surface which might change the optical properties. The tissue samples were kept in an opaque box and moistened with sterile saline solution until the measurement. Tissue preparation, storage and all optical measurements were performed at constant indoor temperature (22 °C) on the day of slaughter with a maximum *ex vivo* time of 6 h.

### 2.2. Experimental Setup

**Figure 1:**
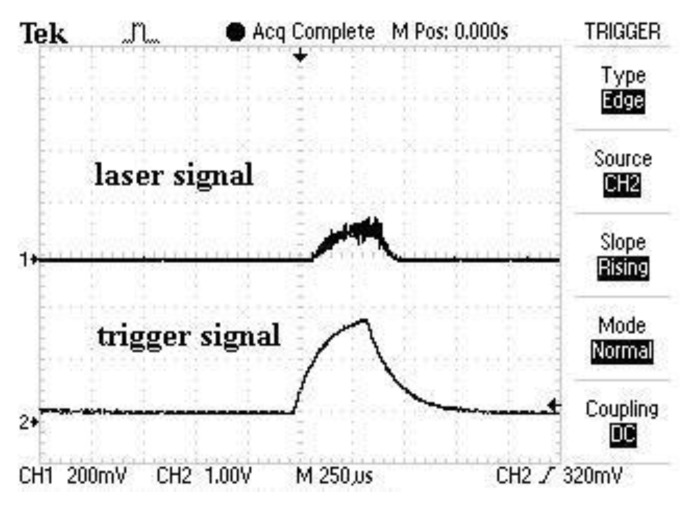
Trigger and laser signal (measured with oscilloscope).

**Figure 2:**
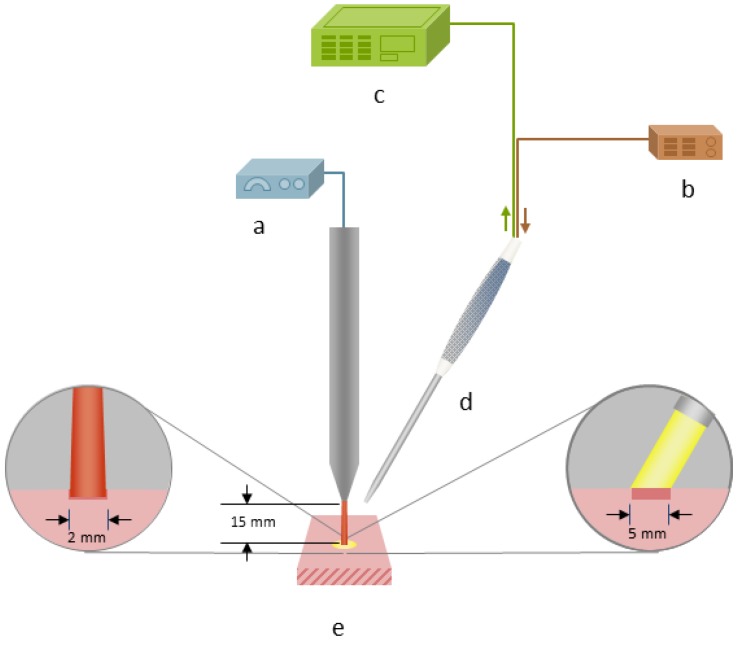
Schematic experimental set-up for laser ablation and optical measurements: (**a**) Er:YAG laser. (**b**) Halogen light source. (**c**) Spectrometer. (**d**) Reflection/backscattering probe. (**e**) Tissue sample (*ex vivo*, pig).

**Figure 3:**
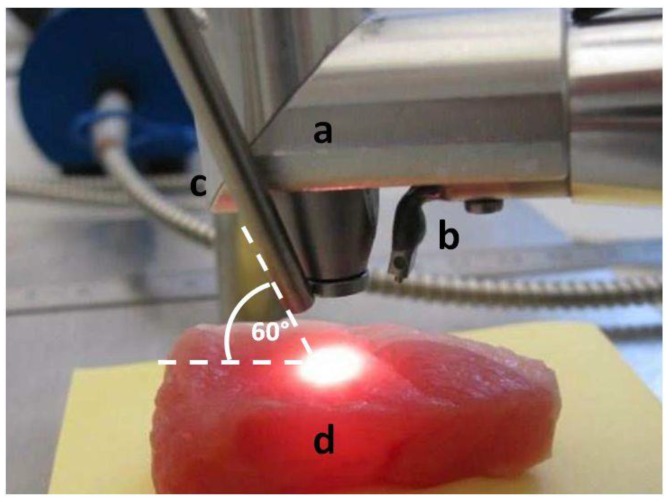
Experimental set-up for laser ablation and optical measurements: (**a**) Hand piece of the Er:YAG laser. (**b**) Internal system for spray cooling. (**c**) Reflection/backscattering probe. (**d**) Tissue sample (in that case muscle tissue, *ex vivo*, pig).

For tissue ablation an Er:YAG laser (2.94 µm, Glissando, WaveLight™, Erlangen, Germany) with a pulse duration of 350 µs, an energy level of 800 mJ per pulse and a pulse frequency of 10 Hz was used. To protect the tissue from thermal damage during laser irradiation, a constant spray water cooling (sterile saline solution, 22 °C room temperature) with a flow rate of 50 mL/min was provided via the internal water cooling system of the laser hand piece. To measure the diffuse reflectance spectra during laser ablation it was necessary to trigger the spectrometer to the laser pulses. The 5 V TTL-signal (transistor-transistor logic) output from the laser served to trigger the spectrometer to the laser pulse. To check the accuracy limit of chronology of the laser pulse and the trigger signal, the signals were tested by using an IR-pulse detector (2–12 μm Lab Bench Detector, Doro Tek GmbH, Berlin, Germany) to detect the laser beam and a power detector (DET210, High Speed Silicon Detector, Thorlabs^®^, Newton, NJ, USA) to detect a VIS laser diode (650 nm) which was triggered by the 5 V TTL-signal output from the laser. Both signals were monitored by an oscilloscope (Figure 1). By this means, it was ensured that at a pulse duration 350 µs with 10 Hz and an integration time of 10 ms the optical measurement happened during the laser pulse. The laser beam was adjusted perpendicularly to the tissue sample surface. The selected distance between the laser hand piece and the tissue was 15 mm. Diffuse reflectance spectra were captured using a reflection/backscattering probe (QR600-7-SR125BX, 200–1100 nm; Ocean Optics^®^, Dunedin, FL, USA) which was fixed 15 mm above the tissue surface. It consisted of six surrounding illumination fibres which emitted the light from the halogen light source (HL-2000, 300–1050 nm; Ocean Optics^®^, Dunedin, FL, USA) and one central collection fibre that gathered the reflected light. All optical fibres had a core diameter of 0.6 mm. To avoid a direct interference with the ablating laser beam, the probe was orientated in an angel of 60° to the tissue surface. This created an elliptic illumination spot being the area of measurement with a transverse diameter of 8 mm and a conjugate diameter of 7 mm. In order to exactly detect the area of laser ablation with a circular diameter of 2 mm prior to each measurement the illumination spot and the ablation spot were precisely adjusted by using a red pilot beam (639 nm) from the laser. In the correct position the spectrometer displayed the highest intensity at this wavelength. For the measurement itself, the pilot beam was turned off. The diffuse reflectance spectra were acquired by a spectrometer (QE 65000, 350–1129 nm; Ocean Optics^®^, Dunedin, FL, USA) linked with a computer working with the software Spectra Suite (Spectra Suite; Ocean Optics^®^, Dunedin, FL, USA). The following software parameters were used: an integration time of 10 ms, a scan to average with 3 and a boxcar of 5. Figure 2 and Figure 3 show the measurement set-up. To meet the requirements for further clinical practicability complete darkness was avoided and the optical experiments were performed in a dimmed environment with residual stray light. Each tissue type was optically measured at ten different spots while performing 30 single measurements per spot. Investigating 15 animals, a total of 27,000 spectra (4500 per tissue type) in a wavelength range from 350–650 nm were recorded for statistical evaluation. To exclude any bias by spot overlapping the distance between the single ablation/measurement spots was chosen to be greater than 5 mm.

### 2.3. Data Processing

High noise occurred in the spectra between 350 nm and 400 nm. Consequently, the focus lay on the 319 data points (after pre-processing) in the range between 400 nm and 650 nm. The distance between the single wavelengths points was 0.8 nm. In order to receive a descriptive diffuse reflectance *R_d_*(*λ*) value the raw signal *S_Rd_*(*λ*) had to be converted. Therefore, a reference spectrum (the light source emission spectrum; by using the reflectance standard WS-1^®^, 250–1500 nm, Ocean Optics^®^, Dunedin, FL, USA) and a background signal *S_D_*(*λ*) were acquired. The background signal *S_D_*(*λ*) was subtracted from the raw signal *S_Rd_*(*λ*) on the one hand and from the light source emission spectrum *S_R_*(*λ*) on the other hand; subsequently, a quotient of both differences was calculated and the diffuse reflectance *R_d_*(*λ*) value was created by multiplying the result by 100%:

$$R_{d}\;\left(\text{λ}\right)=\;\frac{S_{Rd}\left(\text{λ}\right)-\;S_{D}\;\left(\text{λ}\right)}{S_{R}\;\left(\text{λ}\right)-\;S_{D}\left(\text{λ}\right)}\;\times 100\%$$

*S_Rd_*(*λ*) = Diffuse reflectance raw signal

*S_R_*(*λ*) = Light source emission spectrum

*S_D_*(*λ*) = Background signal

*R_d_*(*λ*) = Diffuse reflectance

### 2.4. Statistical Analysis

To estimate the classification quality a leave-one-out cross validation was performed. For each of the wavelength measurement values the mean of all measurements (at each specific wavelength) was subtracted from the measurement value. Thus, a mean value of zero was obtained for the measurements on a wavelength-by-wavelength basis. In order to reduce the number of variables for tissue differentiation a principal component analysis (PCA) was performed. The PCA allows for a decomposition of the data by creating orthogonal and thereby independent linear combinations of the variables, the so-called principal components (PC). There were as many PCs as variables, but the advantage was that only a few were necessary to describe minor than 90% of variation of the data, while the majority of the PCs’ was responsible for less than 1% of the scatter. The relevant principal components were determined in an inner cross-validation. The obtained principal components were classified into one of the tissue types using quadratic discriminant analysis (QDA). In order to evaluate the efficacy of the analysis, the confusion matrix was examined and the overall and tissue specific classification accuracies were calculated. Furthermore, ROC analysis was performed to examine classification performance for all pairwise comparisons of tissues. Calculations were performed using the software package R V2.13.1 [36], with the packages ipred V0.8-11 [37] and Daim V1.0.0 [38].

## 3. Results and Discussion

### 3.1. Results

**Figure 4:**
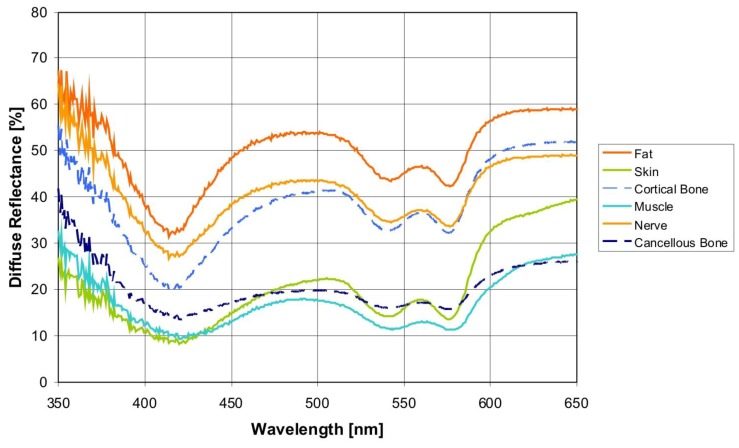
Diffuse reflectance spectra for different hard and soft tissues measured during laser ablation (averaged over 900 measurements per tissue type), spectrometer QE 65000.

**Figure 5:**
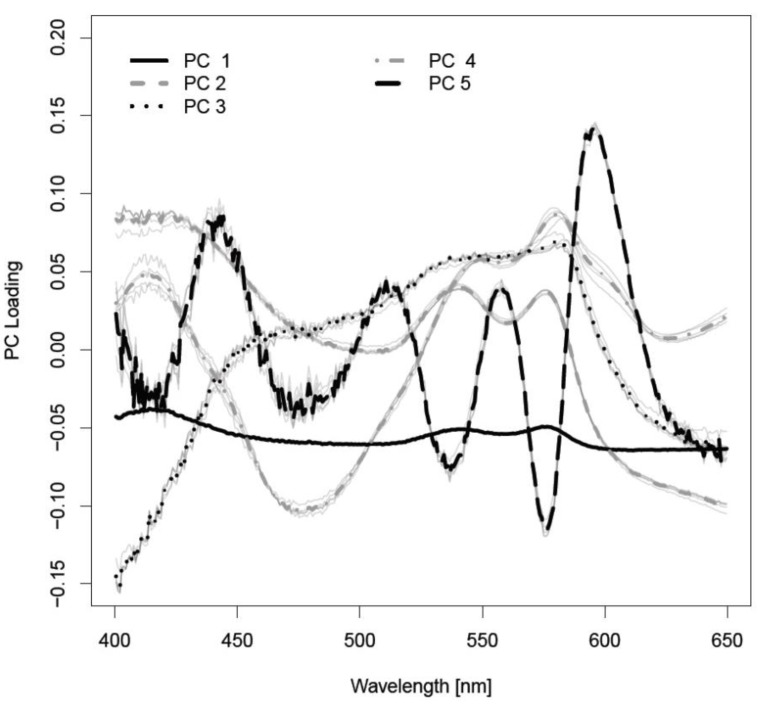
Loadings of the relevant principal components.

The various spectra (Figure 4) were processes by statistical methods to differentiate the spectral curves. 60 principal components (PCs) were considered to be appropriate for tissue differentiation. The first five PCs sufficed (averaged over five cross-validation runs) to determine 99.95% of the variation of the data (see Figure 5). PC1 was responsible for 97.1% of the variance of the diffuse reflectance spectra. PC2 delivered 2.3% of the variance. PC3, PC4 and PC5 contributed 0.4%, 0.1% and 0.03%. The confusion matrix of tissue differentiation is given in Table 1. Nerve tissue was identified with a probability of 83.4% to 99.8%. The differentiation between nerves and cancellous bone reached an AUC-value of 92.4%. The identification of nerve *versus* fat delivered an AUC-value of 83.4%. Areas under the ROC curve (AUC) of all pairwise comparisons between tissues are given in Table 2, sensitivities are shown in Table 3, and specificities are given in Table 4. The classification error was 23.51%.

**Table 1:** Confusion matrix and tissue specific accuracy for the different tissue types.

Tissue	Classified as					
	Cancellous Bone	Cortical Bone	Fat	Muscle	Nerve	Skin
cancellous bone	3375	697	0	2	397	0
cortical bone	2008	2183	75	0	233	1
fat	241	98	3105	0	905	62
muscle	74	3	0	4393	30	0
nerve	176	101	851	0	3401	1
skin	2	313	3	0	53	4129
tissue specific accuracy %	75.5	48.5	70.4	97.6	75.1	91.8

**Table 2:** Tissue differentiation by AUC.

AUC	Cancellous Bone	Cortical Bone	Fat	Muscle	Nerve
cortical bone	0.723				
fat	0.979	0.969			
muscle	0.987	0.999	1.000		
nerve	0.924	0.953	0.834	0.998	
skin	0.998	0.949	0.998	1.000	0.992

**Table 3:** Sensitivity of tissue differentiation.

Sensitivity	Cancellous Bone	Cortical Bone	Fat	Muscle	Nerve
cortical bone	0.533				
fat	0.925	0.939			
muscle	0.979	0.999	1.000		
nerve	0.949	0.971	0.723	1.000	
skin	0.973	0.929	0.998	1.000	0.987

**Table 4:** Specificity of tissue differentiation.

Specificity	Cancellous Bone	Cortical Bone	Fat	Muscle	Nerve
cortical bone	0.824				
fat	0.997	0.976			
muscle	1.000	1.000	1.000		
nerve	0.895	0.940	0.823	0.983	
skin	1.000	1.000	0.978	1.000	1.000

### 3.2. Discussion

Tissue differentiation is an essential part in a closed looped remote feedback system for tissue-specific laser surgery. The general feasibility to differentiate various soft tissue types both pre [31,32] and post laser ablation *ex vivo* [34] and several *in vivo* soft tissue types [35] by diffuse reflectance spectroscopy (DRS) was shown when evaluating this optical method for a remote feedback system by our workgroup. In order to explore the transferability of the system towards a clinical setting it was vital to investigate if tissue differentiation and especially nerve identification is feasible also during the process of laser ablation. If nerve tissue should be protected as effectively as possible, it has to be detected at any time during the ablation. For this purpose, the influence of the laser beam, the spray water cooling, probe angulation of 60° and the alterations evoked by tissue laser ablation (e.g., optical process emissions) had to be considered in the current study. In conformity with the non-contact laser tissue ablation, a remote optical measurement method was chosen. By this means, the alteration of the tissues optical properties by pressure (from the probe) could be avoided [39,40,41,42].

**Figure 6:**
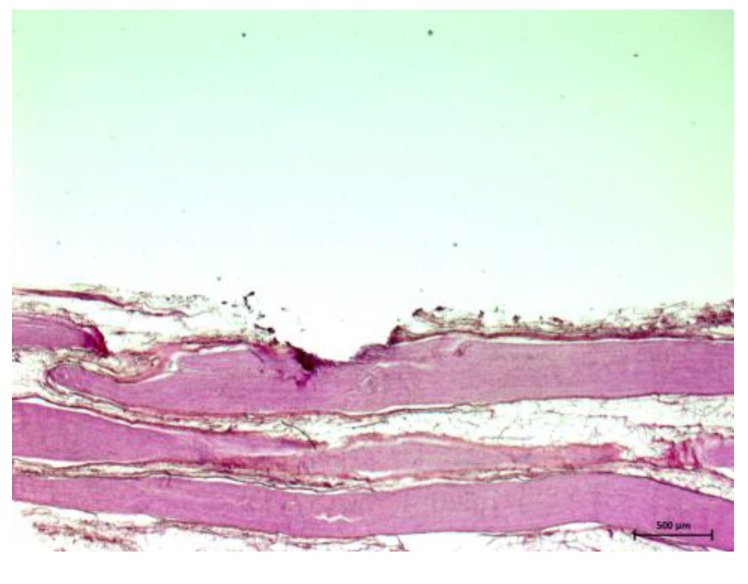
Nerve tissue with laser ablation crater after 10 pulses

**Figure 7:**
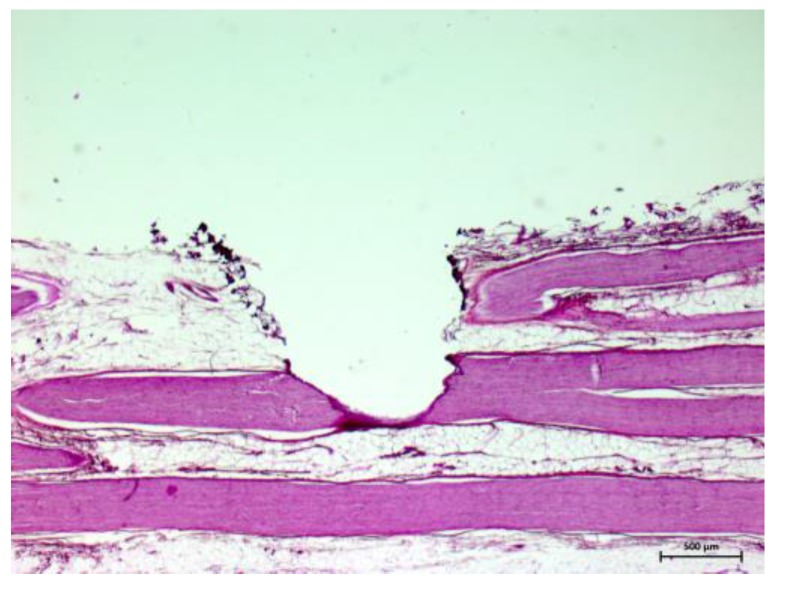
Nerve tissue with laser ablation crater after 20 pulses

The feasibility of tissue identification during laser ablation has been described in literature in brain tissue, using fluorescence spectroscopy and optical enhancers [43,44]. In these works, the optical contrast agent (5-aminolevulinic acid, 5-ALA) was used to induce fluorescence spectra which served to differentiate between malignant and normal brain soft tissue. However, the focus of the present study is DRS-based online tissue identification in the area of head and neck without applied optical enhancers. The acquired spectra occur due to the tissues’ inherent optical properties. Therefore, the application of an optical enhancer—which might be harmful to the patient—was redundant. Prospectively, when regarding laser ablation as a possible method for the sagittal split osteotomy, nerval damage can only be avoided with the aid of an optical feedback system which can operate sufficiently at any time during the laser ablation. Up to now, it was demonstrated that an optical tissue differentiation is feasible prior and after laser tissue ablation. The present, unprecedented work closes the diagnostic loop and shows that nerve can be identified correctly even within the laser pulse. Using diffuse reflectance spectroscopy the tissue pair nerve/cancellous bone was designated correctly with an AUC value of 92.4% (sensitivity: 94.9%, specificity: 89.5%). The classification of nerve tissue and cortical bone yielded a very high AUC value >95%, a sensitivity >97% and a specificity of 94.0%. These findings are of particular importance as the nervous alveolaris runs within the mandible surrounded by the inner cortical layer [17,18]. Compared to a prior study which was carried out without laser ablation [32] the results (AUC: 88.8%, sensitivity: 83.7%, specificity: 78.8%) for these two tissue types are even better in the present study and the outcome is at a similar high level as nerve identification after Er:YAG laser ablation [33]. This observation might be interpreted due to the anatomical structure as a peripheral nerve is surrounded by myelin. Every single nerve fiber is surrounded by thin layer of myelin (epineurium) and a nerve fiber bundle is packed by another myelin sheath (perineurium) [45]. After the myelin sheath was removed by the laser and the nerve fibers were exposed (see Figure 6 and Figure 7). A similar picture emerged for the identification of nerve against fat, which is important in operations of the parotid gland as gland inherits a significant amount of which increases with age. The tissue pair nerve/fat provides a high biological similarity, in particular when regarding the superficial layers of the samples. Both structures contain of up to 75% lipids, e.g., 25% cholesterol and 50% phospholipids [45]. This similarity led to the circumstance that in our prior study the classification performance improved from AUC 75% (sensitivity: 65%, specificity: 75%) before laser ablation to AUC 85% (sensitivity: 88%, specificity: 71%) after laser ablation as the myelin sheath was removed by the laser and the nerve fibers were exposed [34]. These nerve fibers itself could be differentiated more efficient from bone and fat than the superficial myelin. Intriguingly, the current values can be found exactly in between the pre and post ablation accuracies as an AUC value of 83.4%, a sensitivity of 72.3% and a specificity of 82.3% were determined. Additionally, the differentiation of nerve tissue from the soft tissue types muscle and skin were investigated. It was almost perfectly distinguished between muscle (AUC: 99.8%, sensitivity: 100%; specificity: 98.3%) and skin (AUC: 99.2%, sensitivity 98.7%, specificity: 100%).

For the first time, no stable condition but the ongoing process of tissue ablation was examined. In this regard, optical process emissions occurred during laser tissue ablation. Those are luminous phenomena which are connected with the combustion or pyrolysis of organic materials. Especially lipocytes, in fatty and connective tissue and bone marrow, contain a high level of glycerine esters that pyrolyze at temperatures between 300–500 °C. But these light flashes were of short duration (a few ns) compared to the integration time (10 ms) and didn’t hamper the tissue classification considerably. Additionally, the quantity of light flashes was reduced by the spray water cooling [46,47]. Furthermore, laser ablation of biological tissues is known to cause multiple alterations including a change of optical properties [48,49,50] as the ablated tissue area is known to develop a carbonization zone, which scatters and absorbs incident light [51]. The spray water cooling served to minimize this effect, as well. In addition, the Er:YAG laser was chosen for this study as it comes with relatively small alterations, accurately limited lesion edges and low thermal damage [52,53,54]. In combination with—the clinical mandatory—spray water cooling minimal necrosis (≤5 up to 30 µm) and corresponding undisturbed wound healing of bone and soft tissue can be observed as a consequence [52,53,54]. However, the spray water cooling caused increased data variation between 350 and 400 nm. For that reason, this range was faded out and data analysis was performed in a wavelength range from 400 up to 650 nm. In this manner, it was ensured that the water spray cooling did not influence the classification accuracy.

It becomes apparent that the classification performance during laser ablation was only slightly reduced compared to results from prior studies [31,32,33,34]. The setup proved to be appropriate for tissue differentiation during laser ablation. Although the probe was orientated in an angle of incidence of 60° the applied light was diffusely reflected from the tissue in all directions. Thus, probe angulation and distance will influence signal intensity. But we concentrated on spectral shape characteristics for tissue differentiation which are known to be basically determined by tissue parameters. Minor variations of the distance and angulation to the tissue surface are not critical for the method. And the influence of stray light due to this remote detection system has been filtered out by a mathematical algorithm [55]. Neither the laser beam nor the optical process emissions hampered the classification performance substantially. In general, a tissue differentiation was still viable during laser ablation. However, some limitations have to be considered in this study. First, the study was conducted on pigs’ tissue. Compared to other living creatures interspecies differences may show varying results when this method is transferred to other animal models or humans. Second, *ex vivo* tissue is similar but not identical to *in vivo* tissue due to its decreasing moisture and blood content, the missing blood circulation and its progressing de-oxygenation of hemoglobin [56,57]. Hemoglobin is known to be one of the major absorbers in biological tissue. An irreversible alteration of erythrocytes after exposing them to laser energy was reported [48]. Additionally, the specific vascularization of each tissue type may influence the impact of laser light on the tissue. Furthermore, the present experimental setup worked with a probe angulation of 60° and a spray water cooling flow rate of 50 mL/min; it remains to be examined, if a change in the angulation of the probe resp. a different spray condition will impair the results. This also applies to different levels of surrounding stray light. At this, it is necessary to consider the special light conditions in an operation room. Thus, to validate these *ex vivo* results of the present study further research is needed before transferring the results to clinical trials on *in vivo* animal and human tissue. Additionally, the processing time of the employed algorithm for tissue identification which is dependent from computer performance and hardware resources has to be assessed and optimized in order to enable real-time operations. Even though these points have to be considered, this study shows—for the first time—that tissue differentiation based on DRS is feasible during the ablation process. Hence, this is an important step towards the clinical implementation of diffuse reflectance spectroscopy in a closed loop feedback control system for tissue specific laser surgery.

## 4. Conclusions

In order to protect nerves as effectively as possible the tissue has to be identified at any time during laser ablation. A significant classification of the different tissue pairs was demonstrated when performing the measurements within the laser pulses. The applied spray water cooling, the probe angulation of 60° and the optical process emissions did not hamper the differentiation results considerably. Nerve identification by Diffuse reflectance spectroscopy works reliably not only pre and post laser ablation but also during the process of laser ablation. This work is an essential step towards a closed-looped remote feedback system for tissue-specific laser surgery which is able to protect nerve tissue from iatrogenic damage.
